# Moderate-to-Vigorous Physical Activity and Body Composition in Children from the Spanish Region of Aragon

**DOI:** 10.3390/children8050341

**Published:** 2021-04-26

**Authors:** Pilar Ferrer-Santos, Iris Iglesia, Borja Muñiz-Pardos, María Luisa Miguel-Berges, Paloma Flores-Barrantes, Luis A. Moreno, Gerardo Rodríguez-Martínez

**Affiliations:** 1 Instituto de Investigación Sanitaria Aragón (IIS Aragón), 50009 Zaragoza, Spain; pilarferrersantos@gmail.com (P.F.-S.); bmuniz@unizar.es (B.M.-P.); mlmiguel@unizar.es (M.L.M.-B.); pfloba@unizar.es (P.F.-B.); lmoreno@unizar.es (L.A.M.); gerard@unizar.es (G.R.-M.); 2 Growth, Exercise, Nutrition and Development (GENUD) Research Group, Instituto Agroalimentario de Aragón (IA2), University of Zaragoza, 50009 Zaragoza, Spain; 3 Red de Salud Materno Infantil y del Desarrollo (SAMID), Instituto de Salud Carlos III, 28029 Madrid, Spain; 4 Facultad de Ciencias de la Salud y del Deporte, Departamento de Fisiatría y Enfermería, Universidad de Zaragoza, 50009 Zaragoza, Spain; 5 CIBER Fisiopatología de la Obesidad y Nutrición, Instituto de Salud Carlos III, 28040 Madrid, Spain; 6 Departament of Pediatry, Faculty of Medicine, University of Zaragoza, 50009 Zaragoza, Spain

**Keywords:** physical activity, children, body composition, accelerometry, dual-energy X-ray absorptiometry

## Abstract

Most of the studies analyzing the effect of moderate to vigorous physical activity (MVPA) on children’s health do not contain information on early stages or do not use accurate methods. We investigated the association between PA and body composition using objective methods, perinatal data, lifestyle behaviors, and World Health Organization (WHO) physical activity (PA) recommendations. The CALINA study is a longitudinal observational cohort study of children born in Aragon (Spain) in 2009. A total of 308 7-year-old children (52.3% boys) were assessed. We used dual-energy X-ray absorptiometry (DXA) and accelerometry. Rapid weight gain until 12 months and lifestyle behaviors were considered as covariates both in the ANCOVA and linear regression models. A higher percentage of boys met the WHO PA recommendations compared to girls (69.6% vs. 40.9%, respectively; *p* < 0.001). There was a negative association between MVPA and subtotal fat and abdominal fat in both girls and boys. After adjusting for perinatal and lifestyle variables, we found that subtotal body fat, abdominal fat, and fat mass index (FMI) were significantly lower in those classified as active. MVPA was associated with body fat both in boys and girls. More research is needed to identify the cutoffs points of MVPA that generate benefit to boys and girls in all body composition components.

## 1. Introduction

Obesity is a condition characterized by the excess of body fat, affecting both adults and children, and there has been an alarming increase of childhood obesity in the last decades [1]. This is of relevance as both excess body weight and adiposity are associated with a number of comorbidities affecting almost every system in the body, including psychological problems [2]. These comorbidities are in the top leading causes of death in the world [3].

Overweight and obesity prevalence in Spanish school-age children in 2019 were 23.3% and 17.9%, respectively [4]. Maternal smoking, rapid infant growth, or short breastfeeding have shown to be amongst the earliest factors contributing to later excess of body weight or adiposity [2,5]. However, still there is scarce evidence on large cohorts followed since birth to really understand until what extent such factors influence body composition or future health.

Moreover, already during childhood there are several risk factors for overweight and obesity such as those related to lifestyle behaviors including eating habits, low levels of physical activity (PA), or high levels of sedentarism [6,7]. Specifically, previous studies have shown that in school-aged children, high levels of PA are associated with low body fat and high muscle mass content [8], as well as low abdominal fat or low risk of cardiovascular disease risk factors and metabolic syndrome [9].

Due to the important benefits of practicing PA, the World Health Organization (WHO) recommended, for children aged 5–17, a minimum of one hour per day of moderate-to-vigorous physical activity (MVPA). Although this recommendation has shown several benefits [10], only 5% of the official PA recommendations in Spain are based on the WHO’s advice mentioned [11].

Most studies analyzing the influence of PA in children’s body composition do not account for perinatal data, and many of them do not use accurate methods to assess either PA (e.g., accelerometery) or body composition (e.g., DXA). In this, caution should be taken when considering the tool to assess PA levels, as qualitative methods (i.e., questionnaires) have been shown to differ from objective methods (e.g., accelerometery). For example, Rääsk et al. [12] showed that MVPA as assessed with questionnaires was overestimated in less active boys, but underestimated in more active boys, when compared to accelerometery. Nevertheless, and despite being an objective method, accelerometery also has its own pitfalls. A previous study [13] highlighted the functional limitations of these wearable devices depending on the population, stating that comparison between studies are challenging given the different cut-points used. This issue limits the PA interpretation and comparison between studies and need to be considered in studies assessing PA levels. Nevertheless, the use of accelerometery with infant populations has shown great accuracy in previous studies [14,15]. An additional limitation of the existing studies is that they do not appropriately analyze the influence of current WHO PA recommendations in terms of compliance in school-age children with regard to body composition indicators [16,17]. Our aim was to investigate such association objectively in a pediatric population, using accelerometery to assess PA and current WHO recommendations and dual X-ray absorptiometry to assess body composition.

## 2. Methodology

### 2.1. Study Design

This study included data from an observational study named “Growth and Feeding during Early Childhood in Children from Aragón (CALINA)”, which is based on a cohort of children born in the region of Aragon (Spain) in 2009. The initial sample recruited for this project included 1602 subjects [18]. These children were followed every month during the first year of life and every year since then until they reached the age of 7. From September 2016 to September 2017, we contacted all the families recruited in Zaragoza back in 2009 to be re-assessed, requesting them to attend the laboratory located at the University of Zaragoza. A total of 415 families out of 952 agreed to participate, and 339 were finally included in this analysis in terms of the variables required for it (176 boys and 163 girls).

This study was approved by the Ethics Committee in Clinical Research of the Government of Aragon (ref. CP PI13/00105, Spain). The project adhered to the Declaration of Helsinki [19], and all the parents agreed and signed an informed consent form.

### 2.2. Data Collection 

In 2009, the pediatricians of the selected primary care centers collected demographic data including immigrant background, obstetric history including if the mother smoked during pregnancy or not, perinatal history, and BMI of parents at child-birth, among other data [18]. Moreover, as a growth marker of the early postnatal period, we calculated and classified the children between those who were rapid weight gainers and those who were not on the basis of the definition of rapid weight gain (RWG), when a positive change in weight-for-age z-score > 0.67 between birth and 12 months [20]. Weight and recumbent height during the first year of life was measured by well-trained health professionals.

At this stage, demographic factors such as migrant background were also obtained, as well as maternal lifestyle behavior such as smoking habit during pregnancy, which were subsequently used in this analyses as covariates. The election of these variables as covariates were based on the results obtained by a colleague who observed in a recent publication that in our sample, migrant background of the mother, smoking habit during pregnancy, RWG, and maternal and paternal BMI were associated with body composition of children belonging to our study but in a previous follow-up (when they were around 6 years). These variables then, including parental BMI, were used as covariates in our study by assuming that will be also related with their children body composition at the age of approximately 7 years old.

Maternal and paternal BMI were self-reported in the follow-up performed between 2016 and 2017 and also used as covariates in this analysis.

In the 2016–2017 assessment, we collected the following data from the children when they were approximately 7 years-old:

Weekly screen time (WST): Using a previously validated questionnaire, parents reported the number of hours of TV/DVD/video viewing and computer/game console use of their child both for a typical day on weekdays and on weekend days. We summed the reported hours per day on weekdays and weekend days to obtain the total WST (hours in week days + hours in weekend days/7 days per week) [21].

Physical activity (PA): PA was objectively assessed with an Actigraph accelerometer (Actigraph GT3X; Manufacturing Technology Inc. Pensacola, FL, USA). Subjects were asked to wear the belt-like accelerometer on the hip all day during a complete week or at least 3 days per week and 1 day per weekend, recording a minimum of 5 h per day. Children and their parents were instructed to remove the accelerometer only during water-based activities, sleeping, and impact sports, registering duration and reason for removal in a formulary. PA was expressed as average in counts per minute (cpm) and minutes per day of light, moderate, and moderate-to-vigorous PA according to Evenson cut-points [22], with light/moderate PA (101–2295 cpm) and MVPA (≥ 2296 cpm). According to WHO recommendations for PA [23], we classified the sample into two groups: active children, including those who did 60 or more minutes per day of MVPA, and inactive children, those performing less than 60 min of MVPA per day.

Diet: Dietary intake was self-reported by parents through a semi-quantitative food frequency questionnaire (FFQ) [24,25] that had been previously used and validated in the multifactorial evidence-based approach using behavioral models in understanding and promoting fun, healthy food, play, and policy for the prevention of obesity in early childhood study (ToyBox-study). In short, the FFQ consists of a list of foods and beverages with response categories to indicate usual frequency of consumption over the selected time period. We calculated the Diet Quality Index (DQI), which is a largely used index, in cohorts with similar characteristics in order to assess diet in terms of three subcomponents: dietary diversity, quality, and equilibrium [26].

Body composition:

1. Height and weight measurements were measured using a stadiometer with a precision of 1 mm (SECA 225, Germany) and Bioimpedance (BI) (Tanita BC-418, Japan) scale. Determination of z-score values of BMI for age (z-BMI) for girls and for boys was performed using the WHO Anthro Software, according to the WHO growth standards of 2006–2007 [27].
2. Dual-energy X-ray absorptiometry (DXA). DXA scans were performed in a supine position, wearing light clothing with no metal and no shoes or jewelry (21). All DXA scan tests were analyzed by the same researcher using Hologic Explorer scanner and a pediatric version of the software QDR-Explorer, Hologic Corp., software version 12.4 (Bedford, MA, USA). Lean mass (body mass– (FM + bone mass)), percentage body fat mass (percentage of fat grams/total mass). Fat mass index (FMI) was a continuous variable calculated for each participant from data obtained from DXA as fat mass in kilograms/height in square meters. Additionally, fat-free mass index (FFMI) kg/height in meters^2^ was also used in this study [28]. Abdominal adiposity was assessed at a delimited region that was drawn on the digital scan image, delimiting the lower horizontal border on the top of the iliac crest and the upper border parallel to the end of the lowest rib [29].

### 2.3. Statistical Analysis

All analyses were conducted with the SPSS program v22. First, we studied the descriptive characteristics of the sample. Chi squared tests were used to contrast differences between groups in case of categorical variables, and *t*-test or Mann–Whitney test were used for continuous variables depending on the assumption of normality. Statistical significance was set at *p* < 0.05. We studied the differences in body composition variables between two groups: those who met the PA recommendations and those who did not, using *t*-test or Mann–Whitney test.

Linear regression models were performed to check the associations between PA (in minutes of MVPA per week) and different body composition items. Three models were created: the first one without any adjustment, the second model was adjusted by RWG, BMI of the parents, and smoking and migrant status of the mother at birth of the child, as well as the z-score BMI of the children at 7 years. The third model was also adjusted by DQI and WST.

Finally, both in active and inactive children (when they meet or not PA recommendations), we performed an estimation of body composition parameters using analysis of covariance (ANCOVA) models adjusted by z-BMI if they had or did not have RWG, BMI of the parents, smoking habit of the mother during pregnancy, education of the mother, origin of the family, DQI, and WST.

## 3. Results

The characteristics of the sample are shown in Table 1. From the 308 subjects, 161 were boys and 147 were girls. When comparing boys to girls, boys had significantly lower subtotal fat mass and FMI, and significantly higher subtotal lean mass and FFMI (Table 1). Moreover, boys had significantly lower abdominal fat than girls.

According to lifestyle behaviors, boys exhibited a significantly higher screen time use per week than girls of 825 min (CI 763 min–887 min) vs. 728 min (CI 671 min–786 min), respectively (*p* = 0.018), while there were no significant differences for DQI. Moreover, a significantly higher percentage of boys met WHO PA recommendations (69.6% vs. 40.9%, respectively; *p* < 0.001) and achieved higher amount of MVPA per day 73 min/day (CI 69–76) vs. 57.61 min/day (CI 55–61), respectively; *p* < 0.001) than girls. We did not find statistical differences for family origin, RWG during the first 12 months of life, or maternal BMI before pregnancy between boys and girls.

After adjusting by RWG, parental BMI, maternal smoking during pregnancy, origin of the mother, child BMI z-score at 7 years, DQI, and WST, we found that body subtotal fat, abdominal fat, and FMI were all significantly lower both in boys and girls meeting PA recommendations (Table 2). Active boys had significantly lower subtotal fat mass, lower FMI, higher FFMI, less abdominal fat mass, and less abdominal percentage of fat than inactive boys. Active boys also had significantly lower total body percentage of fat. In girls, active individuals had less subtotal fat mass, lower FMI, higher FFMI, less abdominal fat mass, less abdominal percentage of fat, and less total body percentage of fat (Table 2).

Associations between PA and body composition accounting for family, perinatal, postnatal, and lifestyle behavior variables are shown in Table 3 for boys and girls. In all regression models, irrespective of further adjustments, there was a significant negative association between MVPA and subtotal fat mass, abdominal fat percentage, and FMI in both girls and boys. There was also a significant positive association between MVPA and FFMI, and there were no significant association between subtotal lean mass and PA neither in boys nor girls. In models 2 and 3, these associations remained significant after adjusting by RWG, parental BMI, maternal smoking during pregnancy, origin of the family, child BMI z-score at 7 years, DQI, and WST.

## 4. Discussion

As far as we are concerned, this is the first study to assess body composition parameters using DXA in such a high number of 7-year-old children followed up since they were born. The main outcome of this study was that active boys and girls (i.e., the group who met WHO PA recommendations) had lower percentage of fat mass and higher percentage of fat-free mass than their non-active counterparts.

Previous studies showed that higher levels of MVPA are associated with a better body composition in children and a lower cardiovascular risk [10]. These are the main reasons why WHO recommendations include performing at least 60 min of MVPA per day in school-age children [23]. However, there is a limited number of studies examining the benefits of meeting these recommendations in terms of body composition in children or later outcomes.

After adjusting by BMI and perinatal factors, both boys and girls had a significantly negative association between PA and fat mass. This association has been witnessed before in studies using samples with 9–10-year-old children [30]. Moreover, in a 3-year longitudinal study [31], Ara et al. showed that children who regularly participated in at least 3 h per week of sports activities were more prone to avoid total and regional fat mass accumulation. Furthermore, in adolescents participating in the HELENA study [29], it was revealed that MVPA was associated with total and central body fat in adolescents from several countries of Europe. However, this was performed in adolescents not in children.

Our final outcome of interest was to observe differences in body composition on the basis of the fulfillment of MVPA recommendations. We observed both boys and girls in terms of fat mass and lean mass (in girls for lean component only for FFMI). However, other authors, on the basis of a large cohort of European children, observed that current PA recommendations were appropriate for girls but not for boys in terms of reducing cardiovascular risk factors [9]. In this sense, Andersen et al. [32] showed that 85 min of daily PA (rather than 60 min) is likely to be a more appropriate threshold to try reducing cardiovascular risk in boys. Moreover, recently, a very similar publication [33] based on a sample of children from the south of Spain also elucidated that while a low proportion of school-aged children met PA WHO recommendations, a higher proportion of them showed normal weight, no abdominal obesity, and low adiposity in comparison with those who did not meet the recommendations. This is evidence that future studies should elucidate on the effects of lifestyle behaviors including PA, already at early stages, in order to reduce adiposity but also to improve the indices of early cardiovascular risk.

In Canada and the United States, there are no studies analyzing the benefits of accomplishing the PA WHO recommendations in body composition in young children, but there is a further 24-h movement guideline that includes three main recommendations to avoid body composition impairments: 9 to 11 h/night of sleep, ≤2 h/day of screen time, and at least 60 min/day of MVPA. On the basis of the results on a recent systematic review considering these three factors, PA specifically, MVPA was most consistently associated with desirable health indicators, including adiposity, in comparison with the other two [34]. In a study from Román-Viñas [35], the main conclusion was that meeting the 24-h PA recommendation elicited a reduced z-BMI. The same results were observed in adolescents [36], with those who met the WHO recommendations having a lower BMI and lower levels of total and central body fat.

According to the fat-free mass variable, boys showed significantly higher fat-free mass than girls. This difference in body composition between boys and girls might be explained due to a different biological distribution of fat and also because boys usually perform higher levels of PA than girls. In the IDEFICS study [8,21], boys also showed slightly higher mean values in all PA variables except for light PA and inactive time. Moreover, in the same study, the proportion of children who watch TV more than 1 h/day was 29% (33% of males and 25% of females), more evident during weekend days. In previous studies [37], boys had less inactive time but more screen time than girls.

One of the major strengths of this study is that it is based on a longitudinal cohort of children, which is the largest recruited in the region of Aragon in Spain. We had primarily perinatal and family collected data from these children from birth, which strengthens this kind of analysis in children between lifestyle behaviors and body composition. Body composition variables were assessed through anthropometry and also with DXA, which is considered an accurate and precise method especially for body composition measurements. PA data were collected using accelerometers, instead of questionnaires, which is also a more reliable method [22]. Furthermore, the measurements were performed by a trained group of health professionals. Lastly, the fact that we took into account other lifestyle behaviors such as diet or sedentarism to adjust our analyses allowed us to decrease potential bias in our results.

However, this study is not without limitations. The main limitation is that the children included in our sample were selected by convenience and those who accepted at 7 years were perhaps those from families that care about health and give more importance to monitor health-related habits; however, we cannot confirm if there is a sample bias in our results. Moreover, the fact that we restricted the validity of the accelerometer measurements to 5 h per day instead of 8 h as recommended, which was done in order to increase the sample size, might suppose an underestimation of total MVPA, but this bias probably underestimates the power of our results too, but going in the same direction in view of the available literature. However, there might have been cases wherein the same children recorded 5 h as a minimum for some of the days, but others more than that, and previous authors have described the validity of 4 to 5 days as reliable (0.80) as children of these ages varied their day-to-day MVPA less than older children [38]. In this sense, it is also worth mentioning that it would have been of interest to also assess the PA at light levels as it might be more accurate and representative at these ages. However, evidence on the impact of PA of light intensity on adiposity and cardiometabolic risk markers is still not sufficiently supported and controversial [39]. Finally, it is worth mentioning that DXA assessments were not performed in fasting status but on early afternoon during weekdays. There is no evidence in terms of children, but in adults [40], similarly for males and females, it seems that after feeding, independently of macronutrient composition, there is an over estimation of lean mass in detriment of fat mass. However, in this study, assessment was performed just after meals (but not clearly specified what time after), while in our study, a minimum of 2 h was passed.

## 5. Conclusions

Despite the fact that the results are applicable only to a sample of children from a region of northern Spain, these children belong to a cohort that was initially representative (at birth) of this region, and therefore valid in terms of sociodemographic representation. Moreover, the standardized procedures and the well-trained health professionals in charge of the measurements, together with the fact that the obtained results are in line with those obtained in bigger studies, allow us to conclude that, since early ages, it seems that there is a large difference between PA levels between boys and girls, although there is also an important difference in screen time in favor of girls in this case. As early as during childhood, these differences are already remarkable in terms of body composition, specifically in terms of fat mass being higher in girls and lean mass being higher in boys. Moreover, apart from gender differences, it seems that those meeting WHO PA recommendations have a benefit in terms of body composition, especially in total body fat and abdominal fat. Future research questions should address what the barriers for children are in order to meet PA recommendations, especially for girls, so that we can develop intervention studies focusing on propitiating a friendly environment for the practice of MVPA. Finally, it would be ideal to follow these kinds of longitudinal studies until later stages in life in order to better understand the weight of these factors in the development of future chronic diseases.

## Figures and Tables

**Table 1:** Main characteristics of the sample.

	Body Composition				
		Total *N* = 308 Mean (95% CI)	Boys *N* = 161 Mean (95% CI)	Girls *N* = 147 Mean (95% CI)	*p*-Value
Height (m) ^a^		1.26 (1.25–1.27)	1.27 (1.26–1.28)	1.25 (1.24–1.26)	**0.04**
Weight (kg) ^a^		27.40 (26.82–27.96)	27.81 (26.98–28.63)	26.93 (26.15–27.72)	0.34
Subtotal fat (g) ^a^		7.00 (6.68–7.32)	6.55 (6.11–6.99)	7.49 (7.04–7.95)	**<0.001**
Subtotal fat (%) ^a^		29.19 (28.44–29.94)	26.74 (25.79–27.69)	31.87 (30.85–32.89)	**<0.001**
Subtotal lean (g) ^a^		16.29 (15.99–16.59)	17.10 (16.67–17.52)	15.41 (15.03 –15.35)	**<0.001**
BMI (kg/m^2^) ^a^		17.14 (16.88–17.41)	17.19 (16.81–17.56)	17.09 (16.71–17.47)	0.99
z-BMI (kg/m^2^) ^b^		0.71 (1.15)	0.76 (1.26)	0.64 (1.02)	0.52
FMI (kg/m^2^) ^a^		4.36 (4.18–4.56)	4.02 (3.78–4.27)	4.74 (4.48–5.00)	**<0.001**
FFMI (kg/m^2^) ^a^		10.20 (10.08–10.32)	10.57 (10.40–10.74)	9.79 (9.64–9.95)	**<0.001**
Abdominal fat (g) ^a^		355.25 (329.06–381.86)	327.68 (292.28–363.07)	385.46 (346.76–424.16)	**0.001**
Abdominal lean (g) ^a^		1.01 (0.975–1.042)	1.039 (989–1.089)	975.16 (931.30–1019.02)	0.26
Abdominal fat (%) ^a^		24.39 (23.51–25.26)	22.34 (21.28–23.4)	26.63 (25.29–27.96)	**<0.001**
		**Lifestyle Behaviors**			
		**Total *N* = 308** **Mean (95% CI)**	**Boys *N* = 161** **Mean (95% CI)**	**Girls *N* = 147** **Mean (95% CI)**	***p*-Value**
DQI (%) ^a^		81 (80–82)	82 (80–83)	80 (78–82)	0.33
WST (mins) ^a^		779 (736–821)	825 (763–887)	728 (671–786)	**0.018**
MVPA (min/day) ^a^		65 (63–68)	73 (69–76)	58 (55–61)	**<0.001**
Meeting WHO MVPA recommendations		**% (*n*)**	**% (*n*)**	**% (*n*)**	
Yes (active) No (inactive)		55.8 (184) 44.2 (146)	69.6 (119) 30.4 (52)	40.9 (65) 59.1 (94)	**<0.001**
		**Family and Perinatal Factors**			
		**Total *N* = 308** **% (*n*)**	**Boys *N* = 161** **% (*n*)**	**Girls *N* = 147** **% (*n*)**	***p*-Value**
**Family origin ^a^**					
Immigrant		11.4 (35)	8.1 (13)	15 (22)	0.072
Spanish		88.6 (273)	91.9 (148)	85 (125)	
**RWG at 12 months ^a^**					
Yes		35.9 (106)	38.1 (59)	33.6 (47)	0.47
No		64.1 (189)	61.9 (95)	66.4 (93)	
**Smoking during pregnancy ^a^**					
Yes		15.3 (47)	15.5 (25)	15 (22)	1
No		84.7 (261)	84.5 (136)	85 (125)	
		**Mean (CI)**	**Mean (CI)**	**Mean (CI)**	
**Maternal BMI (kg/m^2^) ^a^**		23.69 (23.20–24.19)	24.04 (23.33–24.75)	23.32 (22.62–24.01)	0.118
**Parental BMI (kg/m^2^) ^a^**		25.85 (25.49–26.20)	26.04 (25.54–26.55)	25.64 (25.13–26.14)	0.278

Statistically significant differences (*p* < 0.05) are highlighted in bold. Mann–Whitney test was used for studying differences between boys and girls for non-parametric variables ^a^ (where CI are presented) and *t*-test for parametric variables ^b^ (where SD is presented). The sample for the variable RWG was 295 due to the loss of follow-up of some children in the first year. BMI: body mass index; FMI: fat mass index; FFMI: fat-free mass index; DQI: dietary quality index; WST: weekly screen time; WHO: World Health Organization; MVPA: moderate-to-vigorous PA.

**Table 2:** Adjusted means of body composition parameters between being active or not by sex. Data are adjusted by RWG, BMI of the parents, smoking during pregnancy, migrant origin of the mother, child BMI z-score at 7 years, DQI, and WST.

	**Subtotal Fat Mass (kg)**		**Subtotal Lean Mass (kg)**	
	**Mean**	**SE**	**Mean**	**SE**
**Boys**				
Active	6.322	0.13	17.228	0.188
Inactive	7.149	0.20	16.748	0.283
*p*-value	**0.001**		0.16	
**Girls**				
Active	7.02	0.182	15.631	0.238
Inactive	7.74	0.147	15.138	0.192
*p*-value	<0.01		0.12	
	**FMI**		**FFMI**	
	**Mean**	**SE**	**Mean**	**SE**
**Boys**				
Active	3.87	0.069	10.63	0.057
Inactive	4.40	0.105	10.36	0.085
*p*-value	**<0.001**		**<0.001**	
**Girls**				
Active	4.48	0.09	9.98	0.083
Inactive	4.88	0.08	9.61	0.067
*p*-value	**<0.001**		**0.001**	
	**Abdominal Fat Mass (g)**		**Abdominal Lean Mass (g)**	
	**Mean**	**SE**	**Mean**	**SE**
**Boys**				
Active	309.68	13.50	1048.98	24.15
Inactive	371.75	20.39	1032.74	26.45
*p*-value	**0.013**		0.71	
**Girls**				
Active	347.23	17.99	984.61	30.75
Inactive	400.32	14.54	945.17	24.87
*p*-value	0.025		0.33	
	**Abdominal Fat (%)**		**Total Body Fat (%)**	
	**Mean**	**SE**	**Mean**	**SE**
**Boys**				
Active	21.39	0.45	25.90	0.34
Inactive	24.48	0.68	28.88	0.52
*p*-value	**<0.001**		**<0.01**	
**Girls**				
Active	24.50	0.69	30.21	0.54
Inactive	27.97	0.56	32.94	0.44
*p*-value	**<0.001**		**<0.01**	

Statistically significant differences (*p* < 0.05) are highlighted in bold. SE: standard error; FMI: fat mass index; FFMI: fat-free mass index; DQI: diet quality index; WST: weekly screen time.

**Table 3:** Associations between MVPA and body composition parameters. Each model was adjusted by a number of perinatal and lifestyle behavior variables, as pointed out below.

	Model 1				Model 2				Model 3			
	Boys		Girls		Boys		Girls		Boys		Girls	
	β	*p*-Value	β	*p*-Value	β	*p*-Value	β	*p*-Value	β	*p*-Value	β	*p*-Value
Subtotal fat mass (g)	−0.074	**0.05**	−0.083	**0.037**	−0.092	**0.022**	−0.100	**0.016**	−0.076	**0.061**	−0.104	**0.013**
Subtotal lean mass (g)	0.079	0.14	0.033	0.59	0.090	0.111	0.094	0.15	0.109	0.057	0.10	0.127
Abdominal fat mass (g)	−0.027	0.57	−0.024	0.61	−0.024	0.63	−0.052	0.28	−0.007	0.89	−0.063	0.20
Abdominal lean (g)	0.099	0.10	0.092	0.17	0.108	0.08	0.124	0.09	0.125	**0.05**	0.111	0.13
Abdominal fat (%)	−0.128	**0.01**	−0.129	**0.01**	−0.150	**0.008**	−0.170	**0.001**	−0.143	**0.013**	−0.173	**0.001**
FMI (Kg/m^2^)	−0.108	**0.002**	−0.081	**0.03**	−0.116	**0.002**	−0.109	**0.004**	−0.109	**0.005**	−0.113	**0.004**
FFMI (Kg/m^2^)	0.084	0.06	0.102	**0.05**	0.123	**0.004**	−0.108	**0.005**	0.125	**0.004**	0.160	**0.004**

Model 2: Adjusted by RWG, BMI of the parents, and smoking and migrant status of the mother at birth of the child, BMI z-score of the children at 7 years. Model 3: adjustments of model 2 + DQI and WST. Associations were analyzed by lineal regression in 3 models adjusted by different variables. Statistically significant associations (*p* < 0.05) are highlighted in bold. β corresponds to standardized coefficients. RWG: rapid weight gain; BMI: body mass index; DQI: diet quality index; WST: weekly screen time; FMI: fat mass index; FFMI: fat-free mass index.

## Data Availability

The data presented in this study are available on request from the corresponding author. The data are not publicly available due to data protection issues.
